# Visualisation of coronary venous anatomy by computed tomography angiography prior to cardiac resynchronisation therapy implantation

**DOI:** 10.1007/s12471-018-1132-2

**Published:** 2018-07-20

**Authors:** U. C. Nguyên, M. J. M. Cluitmans, J. G. L. M. Luermans, M. Strik, C. B. de Vos, B. L. J. H. Kietselaer, J. E. Wildberger, F. W. Prinzen, C. Mihl, K. Vernooy

**Affiliations:** 1 Department of Physiology, Cardiovascular Research Institute Maastricht (CARIM), Maastricht University Medical Centre (MUMC+), Maastricht, The Netherlands; 2 Department of Cardiology, Cardiovascular Research Institute Maastricht (CARIM), Maastricht University Medical Centre (MUMC+), Maastricht, The Netherlands; 3 Department of Radiology & Nuclear Medicine, Cardiovascular Research Institute Maastricht (CARIM), Maastricht University Medical Centre (MUMC+), Maastricht, The Netherlands; 4 Department of Cardiology, Radboud University Medical Centre, Nijmegen, The Netherlands

**Keywords:** Coronary veins, Computed tomography angiography, Fluoroscopic angiography, Cardiac resynchronisation therapy

## Abstract

**Background:**

The purpose of this study was to illustrate the additive value of computed tomography angiography (CTA) for visualisation of the coronary venous anatomy prior to cardiac resynchronisation therapy (CRT) implantation.

**Methods:**

Eighteen patients planned for CRT implantation were prospectively included. A specific CTA protocol designed for visualisation of the coronary veins was carried out on a third-generation dual-source CT platform. Coronary veins were semi-automatically segmented to construct a 3D model. CTA-derived coronary venous anatomy was compared with intra-procedural fluoroscopic angiography (FA) in right and left anterior oblique views.

**Results:**

Coronary venous CTA was successfully performed in all 18 patients. CRT implantation and FA were performed in 15 patients. A total of 62 veins were visualised; the number of veins per patient was 3.8 (range: 2–5). Eighty-five per cent (53/62) of the veins were visualised on both CTA and FA, while 10% (6/62) were visualised on CTA only, and 5% (3/62) on FA only. Twenty-two veins were present on the lateral or inferolateral wall; of these, 95% (21/22) were visualised by CTA. A left-sided implantation was performed in 13 patients, while a right-sided implantation was performed in the remaining 2 patients because of a persistent left-sided superior vena cava with no left innominate vein on CTA.

**Conclusion:**

Imaging of the coronary veins by CTA using a designated protocol is technically feasible and facilitates the CRT implantation approach, potentially improving the outcome.

**Electronic supplementary material:**

The online version of this article (10.1007/s12471-018-1132-2) contains supplementary material, which is available to authorized users.

## What’s new?

- Coronary venous anatomy is visualised prior to cardiac resynchronisation therapy (CRT) using a designated computed tomography angiography (CTA) protocol.
- A high concordance (85%) in coronary venous anatomy between CTA and fluoroscopic angiography was found.
- Coronary venous CTA additionally impacted the CRT implantation approach in a quarter of the patients, showing potential for the clinical workup in CRT.

## Introduction

Despite the effectiveness of cardiac resynchronisation therapy (CRT), one-third of eligible patients fail to benefit [1]. Placing the left ventricular (LV) lead in an appropriate position in late activated myocardium remote from scarring is a prerequisite for a response to CRT [2, 3]. The majority of studies investigating LV-lead placement have utilised echocardiography [3] and cardiac magnetic resonance imaging (CMR) [4] to define a LV-lead target segment pre-procedurally. The LV lead was subsequently guided to a coronary vein in the target segment using fluoroscopic angiography (FA). However, the practical issue remains that a target segment cannot be reached intra-procedurally, as not all LV segments are covered by coronary venous anatomy. A recent imaging-guided CRT study demonstrated that in half of the patients, no coronary vein was present in the pre-defined CMR or echocardiographic based target segment. Coronary venous anatomy is highly variable regarding trajectory, shape and number of veins [5, 6]. Imaging is therefore essential and can be used to tailor LV-lead placement by pre-defining a target vein or even considering alternative biventricular pacing options if suboptimal conditions prevail.

FA is most commonly used for visualisation of the coronary veins but is invasive, thus prohibiting pre-procedural anticipation of patient-specific anatomy. Computed tomography angiography (CTA) may enable three-dimensional (3D) non-invasive visualisation of the coronary veins and could be performed prior to CRT implantation.

The purpose of this study was to illustrate the additive value of visualisation of the coronary venous anatomy by CTA prior to CRT implantation by (1) elucidating on clinical cases and by (2) comparing the coronary venous anatomy on CTA with FA.

## Methods

### Study population

Patients referred for a CRT device implantation were prospectively enrolled from April to December 2017 at the Maastricht University Medical Centre. Exclusion criteria were standard contra-indications for CTA including severe renal insufficiency, contrast media allergy and claustrophobia. The institutional Review Board approved the study protocol and patients gave written consent for the use of their data.

### Pre-procedural computed tomography angiography

CTA images were acquired up to 1 month prior to the scheduled CRT implantation. Scans were performed using a third-generation dual-source CT scanner (Somatom Definition Force, Siemens Healthineers, Forchheim, Germany) with a 2 × 192 × 0.6 mm slice collimation; reference tube voltage was set to 100 kV_ref_ using automated tube voltage selection (CARE kV); and a reference quality tube current of 400 mAs using tube current modulation software (CareDose 4D). The gantry rotation time was 250 ms and data were acquired with retrospective gating (pitch: 0.15). Image reconstruction was done with an individually adapted field of view at 0.6 mm slice thickness with an increment of 0.4 mm using a Bv40 kernel and iterative reconstruction (ADMIRE; strength 3). A test bolus was injected at the level of the coronary sinus (CS) or ascending aorta to assess optimal delay for the administration of contrast medium (CM). Delay was calculated by time-to-peak +10 or +20 s for the CS or ascending aorta, respectively. Patients received 72 ml pre-warmed Iopromide 300 (Ultravist, Bayer Healthcare, Berlin, Germany) at a flow rate of 6 ml/s followed by an 84-ml mixed bolus (20% CM/80% saline) and a 40-ml saline flush at a flow rate of 3 ml/s. Total iodine load was 26.6 (21.6 + 5) g. Injection pressure and total amount of CM were monitored by a dedicated information platform (Certegra Informatics Solution, Bayer). Coronary venous anatomy was evaluated under supervision by a radiologist with 7 years’ experience in cardiac CTA. 3D reconstructions of the epicardium and coronary veins were constructed using a semi-automatic segmentation approach (Seg3D v2.4, SCI, University of Utah, USA). A 3D cardiac model comprising the coronary veins and epicardium was interactively shown to the implanting physician prior and during CRT implantation to guide LV-lead placement.

### Intra-procedural fluoroscopic angiography

Coronary venous anatomy was evaluated intra-procedurally by an occlusive FA after placement of the right atrial and right ventricular (RV) lead. The CS was cannulated using a CS guiding catheter (Medtronic, Minneapolis, MN, USA) under FA. After cannulation of the CS, a balloon catheter (Medtronic) was advanced towards the proximal site of the CS for occlusion. Once the balloon catheter was in a stable position, fluoroscopic angiograms of the coronary veins were recorded in right anterior oblique (RAO) and left anterior oblique (LAO) views (Inifinix-I, Toshiba Medical, Ottawara, Tochigi, Japan). The coronary venous anatomy from FA was evaluated together with the implanting cardiologist. After acquisition of the fluoroscopic angiograms, the balloon catheter was withdrawn from the CS and a quadripolar or bipolar LV lead was placed in a coronary vein guided by FA. An electrode with a low voltage threshold without phrenic nerve stimulation and long electrical delay was selected for pacing.

### Data analyses

**Fig. 1:**
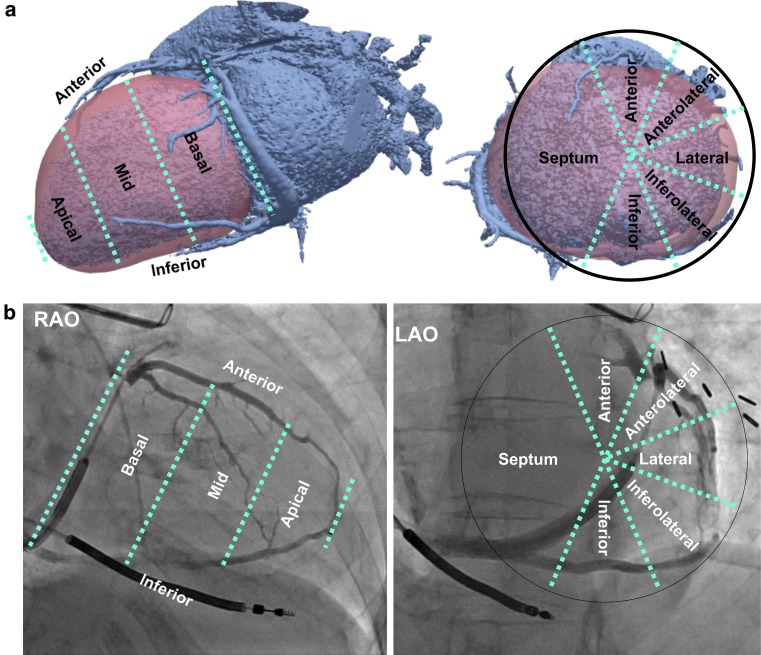
Coronary venous classification used in the present study shown on 3D computed tomography angiography (CTA) images (**a**) and 2D fluoroscopic angiography (FA) images of patient 14 (**b**). *LAO* left anterior oblique, *RAO* right anterior oblique

Continuous variables are expressed as mean ± standard deviation and dichotomous variables in frequencies. The coronary venous angiogram from both CTA and FA was classified according to the American Heart Association 17-segment heart model (Fig. 1). The CS tributaries from the 3D reconstructed CTA images were visually compared with those present on FA in RAO and LAO views (supplementary Fig. I).

## Results

**Fig. 2:**
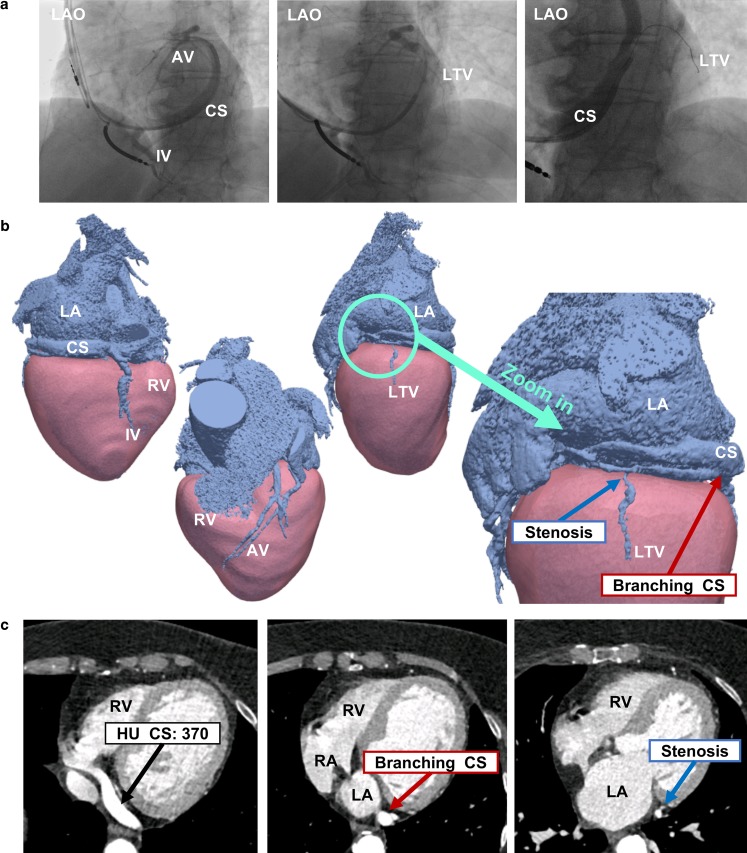
A 78-year-old woman (patient 16) who had undergone an unsuccessful cardiac resynchronisation therapy implantation 5 years earlier as no lateral vein on FA (**a**) was accessible for left ventricular lead placement. CTA in 3D (**b**) and 2D (**c**) revealed a single small and stenotic LTV and CS branching. *AV* anterior vein, *CS* coronary sinus, *IV* inferior vein, *LA* left atrium, *LAO* left anterior oblique, *LTV* lateral vein, *RA* right atrium, *RV* right ventricle

Eighteen patients planned for CRT device implantation were included (Tab. 1). Coronary venous CTA was successfully performed in all 18 patients. CRT implantation was carried out in 15 patients. In one patient CRT was cancelled due to spontaneous recovery of LV ejection fraction, resulting in no indication for CRT according to current guidelines (patient 9) [1]. One patient (patient 16, Fig. 2) was initially planned for CRT device implantation but was treated with a dual-chamber pacemaker after suboptimal coronary venous anatomy on CTA. This patient had undergone an unsuccessful CRT implantation 5 years earlier as no lateral vein (LTV) was accessible for LV-lead placement. The patient was referred for reconsideration of CRT. CTA imaging revealed two major issues for conventional LV-lead placement (Fig. 2). First, there was only a single small LTV available. Second, the CS branched into two sub-veins, further complicating cannulation of the single vein. For these reasons, CRT implantation was abandoned and the patient was treated with a dual-chamber pacemaker only instead. One patient (patient 15) died prior to the scheduled CRT implantation, unrelated to the study protocol.

**Table 1:** Patient characteristics

Patient characteristics (*n* = 18)	
*Demographics*	
Age (years)	69 ± 9
Male (*n*)	13 (72%)
BMI (kg/m^2^)	29 ± 5
Ischaemic cardiomyopathy (*n*)	12 (67%)
NYHA (II/III)	7/11
*CMR LV function*	
LVEF (%)	27 ± 9
EDV (ml)	302 ± 86
ESV (ml)	234 ± 93
SV (ml)	69 ± 18
*ECG characteristics*	
Sinus rhythm (*n*)	14 (78%)
Atrial fibrillation (*n*)	3 (17%)
Paced rhythm (*n*)	1 (6%)
QRS duration (ms)	145 ± 24
LBBB (*n*)	9 (50%)

*BMI* body mass index, *CMR* cardiac magnetic resonance, *ECG* electrocardiogram, *EDV* end-diastolic volume, *ESV* end-systolic volume, *LBBB* left bundle branch block, *LV* left ventricular, *LVEF* LV ejection fraction, *NYHA* New York Heart Association, *SV* stroke volume

In one patient (patient 5, supplementary Fig. II) CRT implantation was postponed due to the finding of a RV thrombus on CTA for which anticoagulant therapy was initiated. The patient still underwent CRT implantation 6 months post-dissolution of the RV thrombus on repeat CTA.

**Fig. 3:**
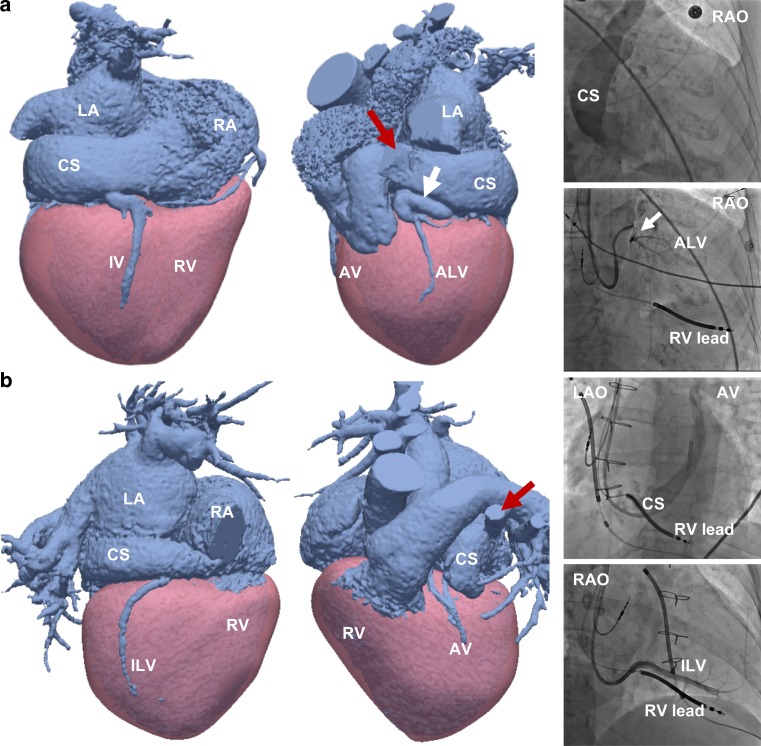
CTA and FA of a 77-year-old woman (patient 4, **a**) and a 49-year-old woman (patient 10, **b**) with a left-sided persistent superior vena cava (*SVC*, *red arrow*) and CS dilatation. Note the sharply angulated vein (*white arrow*) in patient 4 (**a**) and the limited coronary venous anatomy in patient 10 (**b***)*. *ALV* anterolateral vein, *AV* anterior vein, *CS* coronary sinus, *ILV* inferolateral vein, *IV* inferior vein, *LA* left atrium, *LAO* left anterior oblique, *RA* right atrium, *RAO* right anterior oblique, *RV* right ventricle

A standard left-sided CRT implantation approach by entering the left subclavian or cephalic vein and advancing the CRT leads to the right atrium through the left innominate vein was used in 13 patients. A right-sided implantation approach was carried out in the remaining 2 patients (numbers 4 and 10, Fig. 3) because of a persistent left-sided superior vena cava (SVC) with no communicating left innominate vein to reach the right-sided SVC from the left subclavian vein. In patient 10, the presence of a persistent left-sided SVC was known beforehand, and in patient 4 it was found on coronary venous CTA. Both patients had dilatation of the CS as a consequence of the persistent SVC.

**Fig. 4:**
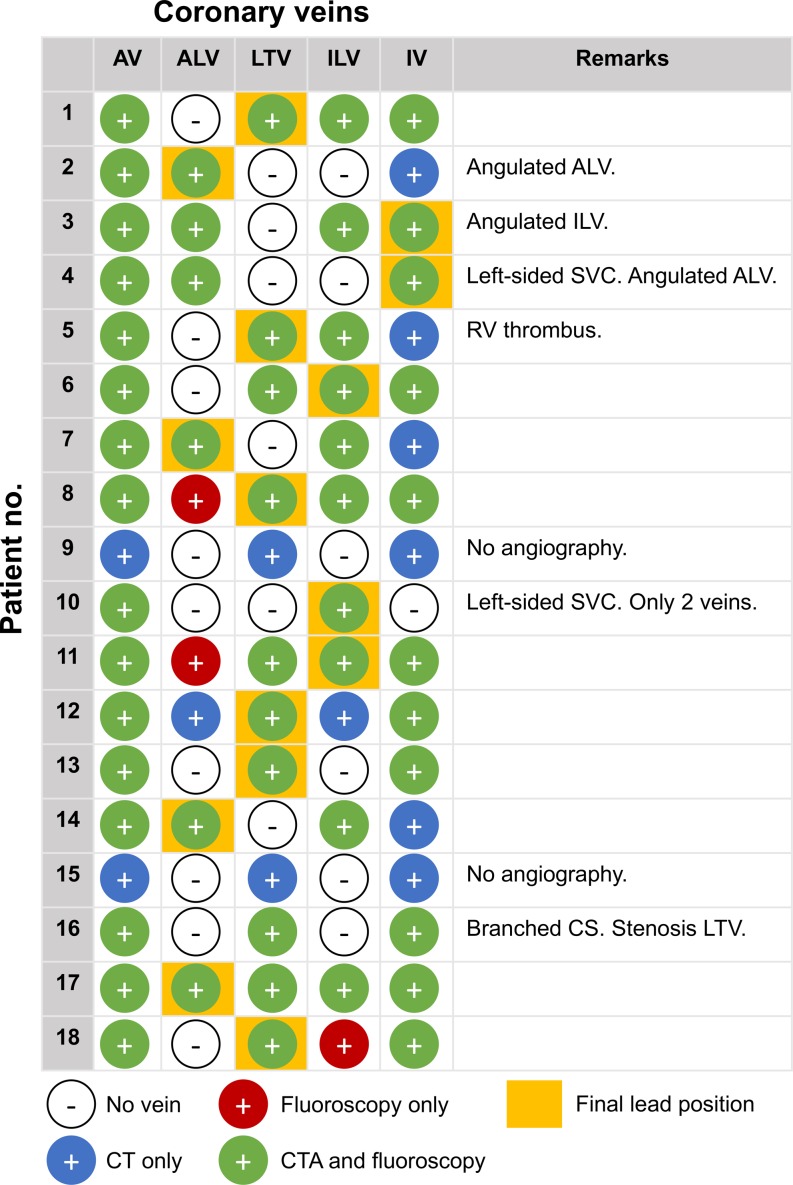
Coronary veins on CTA and FA with final left ventricular lead position. *ALV* anterolateral vein, *AV* anterior vein, *CS* coronary sinus, *ILV* inferolateral vein, *IV* inferior vein,* LTV* lateral vein, *RV* right ventricle, *SVC* superior vena cava

An overview of the coronary veins present on CTA and FA with final LV-lead placement per individual patient is provided in Fig. 4. In one patient (patient 4, Fig. 3a) the LV lead was initially targeted towards the anterolateral vein, but cannulation did not succeed due to a steep angulation at the ostium. After multiple failed attempts, the LV lead was finally placed in the inferior vein (IV). A sharp angulation of the initial target vein (inferolateral vein (ILV)) was also the case in patient 3 (supplementary Fig. III), where cannulation failed and the LV lead was finally cannulated into the IV and advanced up to the ILV through an anastomosis.

The number of veins per patient ranged between 2 and 5. Coronary venous anatomy was extremely limited to only two veins in patient 10 (Fig. 3b). A total of 62 veins from 16 patients with both CTA and angiographic images were visualised. Eighty-five percent (53/62) of the veins were visualised on CTA as well as FA, while 10% (6/62) were visualised on CTA only, and 5% (3/62) were visible on FA only. A total of 22 veins were present on the lateral or inferolateral wall, of which 95% (21/22) were visualised by CTA. Veins visualised on CTA only were most often located inferiorly (4/6), while veins visualised on FA only were small and located anterolaterally (2/3) or inferolaterally (1/3).

## Discussion

This study illustrates the additive value of coronary venous imaging by CTA prior to CRT implantation by elucidating clinical cases and by comparing coronary venous anatomy visualisation by CTA with FA.

In patients with both CTA and FA data, there was a high concordance (85%) between coronary venous CTA and FA. Interestingly, 10% of the veins were visualised by CTA but not on FA, indicating that CTA may even have the capability of identifying veins that are not shown by FA. Two-thirds of the veins missed by FA were, however, located inferiorly, which is usually not the preferred site of LV-lead placement. In contrast, 95% of the ILV or LTV (the preferred veins in CRT) were visualised by CTA. Five per cent of the veins were only visible on FA and therefore missed on CTA. These three veins were, however, small and not suitable for LV-lead placement.

### The relevance of coronary venous anatomy visualisation in CRT

In about a quarter of our patients, pre-procedural cardiac venous CTA had a major impact on the CRT implantation approach. In two patients, a right-sided implantation was carried out due to a persistent left-sided SVC with no left innominate vein. In one patient, implantation was postponed for 6 months due to a RV thrombus, and in another patient, a dual-chamber pacemaker was implanted instead of CRT due to unfavourable coronary venous anatomy.

Coronary venous CTA was particularly useful for the implanting physician by visualising the coronary veins in 3D prior to CRT implantation, allowing anticipation of patient-specific anatomy. The number and trajectory of coronary veins was highly variable in our patients; in two of them, for instance, initial target veins were inaccessible during implantation due to steep angulations at the ostium, and in one patient only two coronary veins were present with limited options for conventional LV-lead placement. A high variability in coronary venous anatomy was also seen in coronary venous CTA imaging in 121 perfusion-fixed post-mortem human hearts [7] Remarkably, in 29% of the hearts no coronary vein was present in the inferolateral region, which is often the site of late activation and subsequently a sweet-spot for LV-lead placement in CRT.

Besides CTA, coronary venous anatomy can also be visualised pre-procedurally in a subset of CRT candidates who are already scheduled for a routine coronary angiography using venous phase coronary angiography [8] Coronary venous anatomy visualisation may be useful in identifying those patients with suboptimal coronary venous anatomy and may potentially simplify the CRT implantation procedure, as it may reduce the need for extensive fluoroscopic coronary venous angiograms. In addition to CRT, evaluation of the coronary venous anatomy may also be relevant for other procedures, including radiofrequency ablation [9] or cell therapy [10].

### Moving towards a tailored coronary venous CTA protocol

The coronary venous anatomy has been assessed by a 4-MDCT scanner and compared with FA in a small study of seven patients [11] but still remains an underexposed area of research compared to the arterial system. The greatest challenge for coronary venous imaging is determining optimal delay for CTA acquisition after the administration of CM, whilst still keeping the total iodine load low. Recent coronary arterial CTA studies demonstrate that CM protocols tailored to the individual patient lead to robust enhancement patterns and reduction of the amount of CM used [12, 13]. CRT patients have low LV function, resulting in prolonged duration for CM to reach the coronary system. Contrast medium furthermore circulates in the body, is diluted by blood, and the bolus disperses as it moves downstream in the circulatory system. The coronary veins are located more distal from the injection site in comparison with the arteries and therefore encounter more dilution. In addition, CM-enhanced blood recirculates and may contribute to the overall pattern of contrast enhancement achieved on CTA imaging. In our coronary venous CTA protocol contrast and thus high attenuation of the coronary veins was accomplished by accurate timing of CM administration (+10 s for the CS; +20 s for the ascending aorta) using a relatively low dose of CM (26.6 g iodine in total).

### Future prospects

Stimulating the correct LV pacing site remains a critical factor for response to CRT. The need for sites of late activation remote from scarring, acceptable pacing thresholds without phrenic nerve stimulation, limited and suboptimal coronary venous anatomy places major constraints on the available LV pacing sites. In case of limited coronary venous anatomy or extensive scarring around the veins, alternative approaches for biventricular pacing could be considered, including endocardial pacing [14, 15], trans-septal pacing [16] or epicardial pacing using a minimally invasive video-assisted thoracic surgical approach [17].

In this study, we focussed primarily on coronary venous anatomy imaging. However, additional advantage could be taken from CT in the context of CRT by incorporating wall thinning, wall motion [18] or delayed enhancement as a metric of myocardial scarring. Imaging of coronary venous anatomy together with dyssynchrony metrics and myocardial scarring using only CT prior to CRT was very recently performed in 54 patients by Truong et al. [19]. This is particularly relevant in patients with already existing pacing systems [20] in which CMR is contra-indicated. An additional potential advantage of CTA may be visualisation of the phrenic nerve [21] in relation to the coronary venous anatomy, to avoid phrenic nerve stimulation around the LV lead.

### Limitations

This is a single-centre study using a limited number of patients. However, coronary venous CTA already impacted on a substantial percentage of the CRT implantations, indicating that CTA prior to CRT may be of important clinical value. CM should be used with caution in patients with severe kidney dysfunction; however CM doses were kept low.

## Conclusion

Visualisation of the coronary venous anatomy by CTA is representative of the anatomy recorded intra-procedurally under FA and has additive value for CRT implantation. CTA allows patient-specific anticipation of coronary venous anatomy and determination of relevant LV-lead pacing sites, making it a diagnostic of high potential in CRT.

## Caption Electronic Supplementary Material

Electronic supplement with two additional figures. Figure I contains representative 3D CTA-images and FA images of patient 1. Figure II contains 2D CTA-images of patient 5 demonstrating the RV thrombus from transversal and coronal view.
