# Supplementary material for: Visualisation of coronary venous anatomy by computed tomography angiography prior to cardiac resynchronisation therapy implantation
Source: Neth Heart J. 2018 Jul 20;26(9):433–44. doi: 10.1007/s12471-018-1132-2 (PMC6115304; doi:10.1007/s12471-018-1132-2)

**Supplement**

**Figure I**

3D CTA-images (A) compared with the FA (B) of a 69-year old male (patient 1). AV=anterior vein, ALV=anterolateral vein, CABG=coronary artery bypass graft, CS=coronary sinus, ILV=inferolateral vein, IV=inferior vein, LA=left atrium, LAO=left anterior oblique, LTV=lateral vein, RA = right atrium, RAO=right anterior oblique, RV=right ventricle.

**
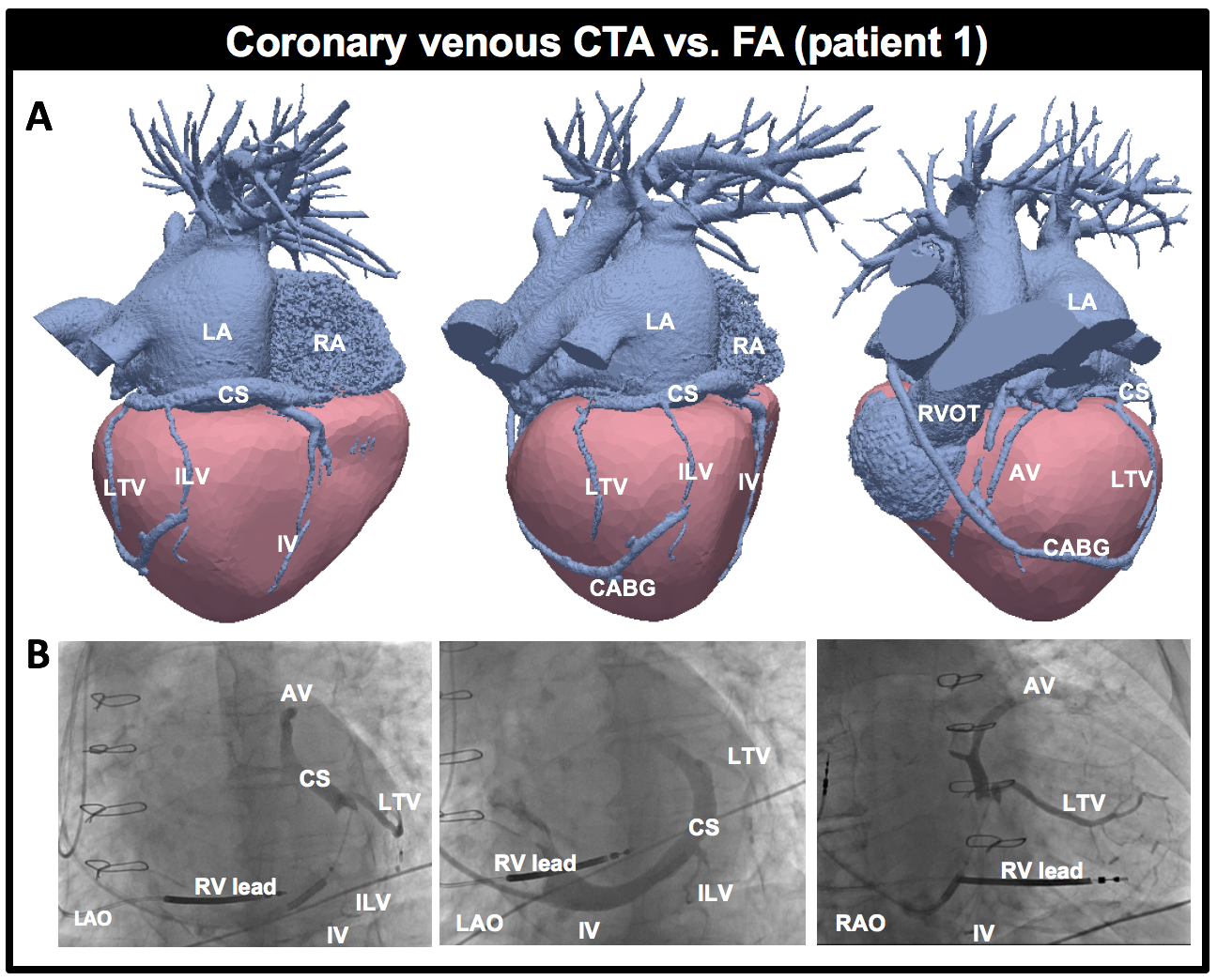
**

**Supplement**

**Figure II**

Transversal (A) and coronal (B) image of a 59-year old woman (patient 5) who demonstrated a RV thrombus of 1x2cm size (red arrow) on CTA. The abnormalities shown on the apex of the LV (green arrow) resulted from LV aneurysmectomy a few years earlier. LV=left ventricle, RV=right ventricle.

**
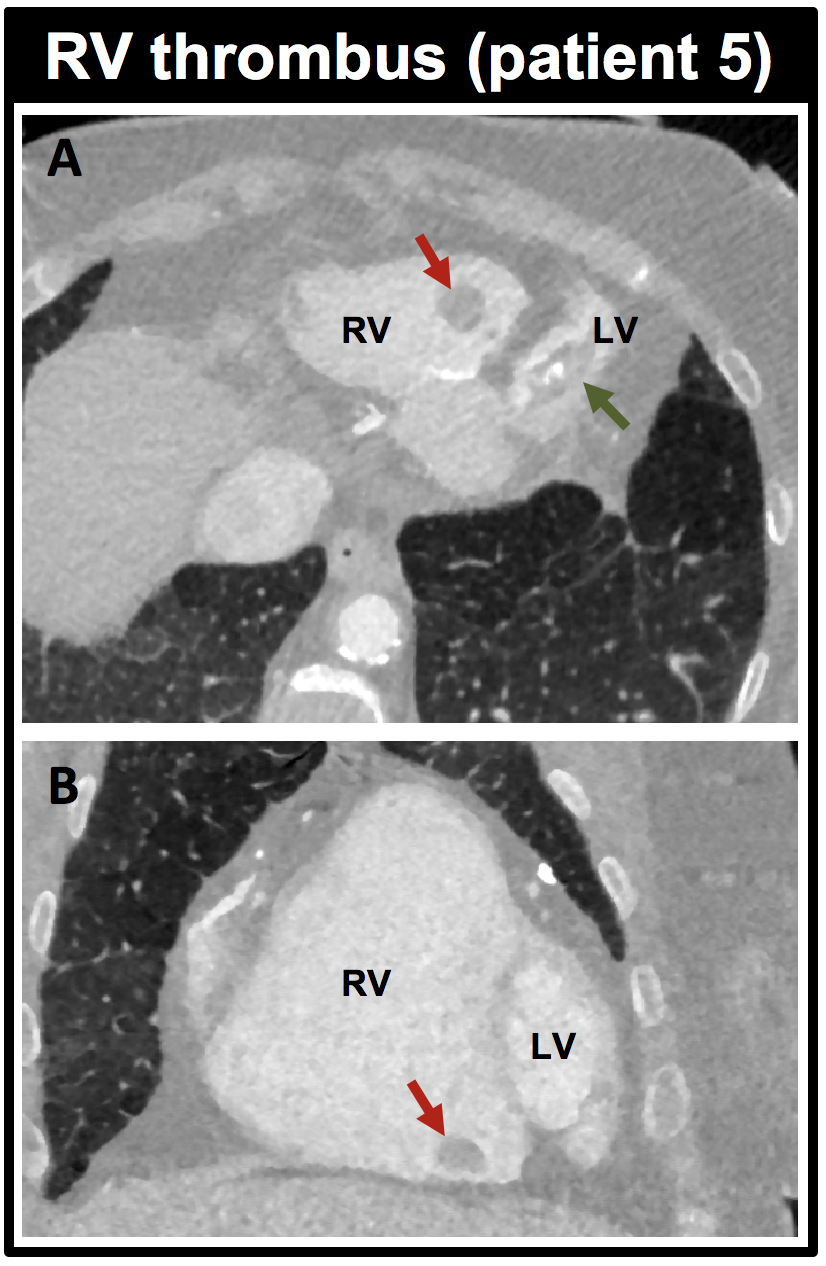
**

**Supplement**

**Figure III**

FA of a 73-year old male (patient 3) with anastomosis (green arrow) between the ILV and IV. Cannulation of the ILV was unsuccessful due to the sharp angulation (red) at the ostium. The LV-lead was finally advanced towards the ILV by cannulation of the IV. CS=coronary sinus, ILV=inferolateral vein, IV=inferior vein, LAO=left anterior oblique, LV=left ventricular, RAO=right anterior oblique, RV=right ventricular.


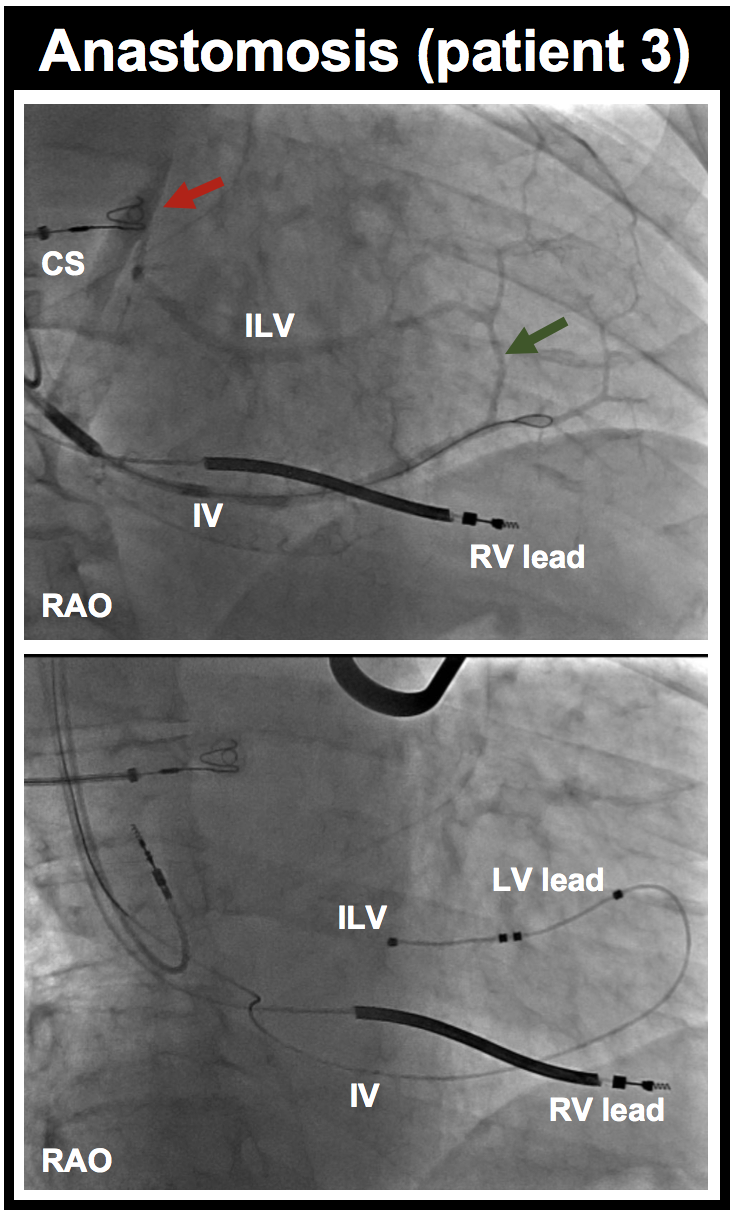

Supplement: Supplementary file 1 — Electronic supplement with two additional figures. Figure I contains representative 3D CTA-images and FA images of patient 1. Figure II contains 2D CTA-images of patient 5 demonstrating the RV thrombus from transversal and coronal view. [file 12471_2018_1132_MOESM1_ESM.docx]
